# Numerical Investigation of Flow Characteristics in the Obstructed Realistic Human Upper Airway

**DOI:** 10.1155/2016/3181654

**Published:** 2016-09-20

**Authors:** Xingli Liu, Weiwei Yan, Yang Liu, Yat Sze Choy, Yikun Wei

**Affiliations:** ^1^College of Metrology and Measurement Engineering, China Jiliang University, Hangzhou, China; ^2^Department of Mechanical Engineering, The Hong Kong Polytechnic University, Kowloon, Hong Kong; ^3^Faculty of Mechanical Engineering and Automation, Zhejiang Sci-Tech University, Hangzhou, China

## Abstract

The flow characteristics in the realistic human upper airway (HUA) with obstruction that resulted from pharyngeal collapse were numerically investigated. The 3D anatomically accurate HUA model was reconstructed from CT-scan images of a Chinese male patient (38 years, BMI 25.7). The computational fluid dynamics (CFD) with the large eddy simulation (LES) method was applied to simulate the airflow dynamics within the HUA model in both inspiration and expiration processes. The laser Doppler anemometry (LDA) technique was simultaneously adopted to measure the airflow fields in the HUA model for the purpose of testifying the reliability of LES approach. In the simulations, the representative respiration intensities of 16.8 L/min (slight breathing), 30 L/min (moderate breathing), and 60 L/min (severe breathing) were conducted under continuous inspiration and expiration conditions. The airflow velocity field and static pressure field were obtained and discussed in detail. The results indicated the airflow experiences unsteady transitional/turbulent flow in the HUA model under low Reynolds number. The airflow fields cause occurrence of forceful injection phenomenon due to the narrowing of pharynx caused by the respiratory illness in inspiration and expiration. There also exist strong flow separation and back flow inside obstructed HUA owing to the vigorous jet flow effect in the pharynx. The present results would provide theoretical guidance for the treatment of obstructive respiratory disease.

## 1. Introduction

The inhaled particles are the culprit for aggravating fog and haze pollution, which seriously jeopardizes the human health. The human upper airway (HUA), which consists of oral cavity, nasal cavity, pharynx, larynx, and trachea, is the main passageway for inhaled particles damaging human health, resulting in respiratory diseases range from simple common cold, snoring, and obstructive sleep apnea (OSA) to more serious pneumonia and even lung cancer [1, 2]. To thoroughly understand the airflow dynamics and particles deposition pattern, it is of great significance to investigate the internal airflow characteristics within the HUA.

To date, many experimental studies have been carried out to explore airflow properties in the laboratory HUA models. Girardin et al. (1983) applied the LDA measurements to determine the velocity field in a human nasal cavity model made of cadaver at five coronal sections [3]. Heenan et al. (2003) studied the airflow field in an idealized representation of human oropharynx (HOP) via particle image velocimetry (PIV), which clearly indicated the complex nature of HOP flow, with three-dimensionality and several regions of separation and recirculation evident [4]. Based on the hot-wire anemometry and flow visualization methods, Johnstone et al. (2004) measured the axial velocity field in the central sagittal plane of an idealized representation of human extrathoracic airway (ETA) [5]. They found that the airflow patterns in the ETA were rather complex, which involved several regions of separated, secondary, and recirculating flow. Doorly et al. (2008) investigated airflow characteristics in the fabrication of a replica model of nasal cavity by the PIV and flow visualization [6]. Quantitative details on the variations of velocity in different regions of nasal cavity were obtained. Recently, Kim and Chung (2009) extended their work from Kim and Chung (2004), where tomographic PIV measurements were taken in a full HUA model under periodic flow condition [7, 8]. The velocity distributions in both sagittal and coronal planes were obtained. The results suggested that the main stream went through the backside of larynx and trachea in inspiration and the frontal side in expiration.

Although the experimental measurements are manifestly helpful in understanding the airflow dynamics in the HUA, the lack of complete fidelity of hot wire and PIV has been clearly demonstrated [5, 9]. On the other hand, the CFD is now becoming a strong tool not only to analyze the airflow patterns, but also to get the variations of temperature, pressure, and wall-shear stress in the HUA [10, 11]. In recent years, the complex representative and realistic HUA models have been reconstructed. Wang et al. (2009) built the HUA based on the CT images of a healthy volunteer to quantitatively study the relationship between airflow structure and function [12]. It was observed that the air mainly passed through the floor of nasal cavity in the common, middle, and inferior nasal meatus, and the higher airway resistance and wall-shear stress were found on the posterior nasal valve. Mihaescu et al. (2008) applied the LES to depict the transitional/turbulent airflow field in the human realistic pharyngeal airway with obstruction, which revealed that the LES can provide an increased level of detail and accuracy for unsteady, separated, or vortical turbulent flow situations [13]. Likewise, Lu et al. (2014) calculated the pressure and shear stress distribution in the realistic HUA with OSA before and after treatment by the LES [14]. There existed a significant pressure and shear stress dropping region near the soft palate before treatment, and the flow resistance in the upper airway was decreased and the wall-shear stress value was significantly reduced after treatment. Recently, Wang and Elghobashi (2014) developed a direct numerical simulation-lattice Boltzmann method (DNS-LBM) to investigate the airflow in two patient-specific HUAs reconstructed from CT-scan data under inspiration and expiration [15]. The numerical results showed that the DNS-LBM solver can be used to obtain accurate airflow details and it was a powerful tool to locate the obstruction in the HUA.

In the previous studies, the inspiration and expiration behaviors in both experiments and simulations were mostly separately performed, resulting in discontinuities of inspiration and expiration in respiratory cycle. The respiratory intensity was also mostly assumed to be constant, which could also import discrepancy with the real respiratory process. Although the airflow patterns in the HUA considered of time-dependent respiratory intensity have been reported [12, 16], the airflow dynamics in the HUA is still far from being completely understood. Therefore, we aim at numerically studying airflow dynamics in the realistic obstructed HUA under continuous inspiration and expiration with varied respiratory intensities. The present study would provide a theoretical guidance for the treatment of respiratory disease with obstruction in the HUA.

## 2. Methods

### 2.1. Problem Description

**Figure 1:**
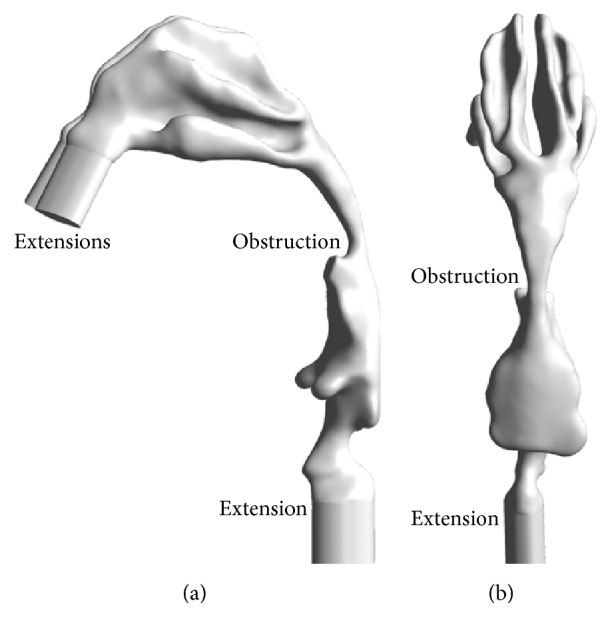
The obstructed HUA model: (a) sagittal view; (b) dorsal view.

Based on the medical CT-scan images of a Chinese male patient (38 years, BMI 25.7), the 3D anatomically accurate HUA model with obstruction resulting from pharyngeal collapse was reconstructed using the image processing software Mimics, as illustrated in Figure 1. The images were obtained in the axial plane with a resolution of 0.7 × 0.7 mm^2^, and the slice thickness is 0.625 mm. To implement the inlet and outlet boundary condition, two extensions were added on the nostrils, and another extension was added on the downstream region of larynx. During inspiration, the extensions on two nostrils were set as the inlet velocity boundaries, and the extension on the downstream region of larynx was set to be the outlet pressure boundary. During expiration, the boundary conditions were contrary to the inspiration process.

To design continuous inspiration and expiration, the numerical results of airflow field at the end of inspiration were imposed to be the initial state of expiration, and the simulation outcomes of airflow field at the end of expiration were designed to be the next initial state of inspiration. In our simulations, the variation of the inlet velocity with respect to the time was set to be periodic sine function; namely,

(1) $$\begin{array}{c} u=\frac{Q}{A}\sin\left(\;\frac{2\pi }{T}t\;\right), \end{array}$$

where *u* is the respiratory velocity, *Q* is the respiratory intensity, *T* is the time of a complete respiratory cycle, and *A* is the inlet cross-sectional area, which represents the area of nostril in inspiration and the area of trachea in expiration.

### 2.2. Numerical Method

The software of CEM-CFD was applied to mesh the obstructed HUA model. Due to the complexity of respiratory structure, the meshes were generated by adopting the tetrahedral elements. To accurately calculate the airflow properties, a refined mesh was employed near the pharynx and larynx. The calculation results of velocity were mesh-convergent to within a prescribed tolerance (~0.2%).

After meshing, the CFD software package Fluent 15.0 was applied to solve the flow governing equations for modeling airflow in the suffering HUA. The time step of calculation was 0.001 s, and the number of time steps was 2500 for individual inspiratory and expiratory airflow. The inlet velocity was designed to be periodic sine function, which has been defined in (1), by the user-defined function (UDF). Since the LES is a validated method for capturing transitional/turbulent unsteady, separated, or vortical flows accuracy [17], consequently it was applied to reveal such relevant flow features in the flow separation region located near the minimum cross-sectional area of the airway and the downstream region. In the LES approach, the filtering operation for a variable (*x*) can be expressed as

(2) $$\begin{array}{c} \overset{-}{\phi }\left(\;x\;\right)=\frac{1}{V}\int_{V}\phi \left(\;x^{\prime }\;\right)G\left(\;x,x^{\prime }\;\right)dx^{\prime }, \end{array}$$

where *V* is the volume of a computational cell and the filter function *G*(*x*, *x*′) is defined as

(3) $$\begin{array}{c} G\left(\;x,x^{\prime }\;\right)=\left\{\begin{array}{cc} 1, & for\;\;x^{\prime }\in V,\\ 0, & otherwise. \end{array}\right. \end{array}$$

The filtering process effectively filters out eddies whose scales are smaller than the filter width or grid spacing, and the filtered Navier-Stokes equations for mass and momentum conservation can be written as

(4) ∇·u−=0,ρ∂u−∂t+ρu−·∇u−=−∇P−+μeff∇2u−,

where $\overset{-}{u}$ is the filtered velocity, $\overset{-}{P}$ is the filtered pressure, *t* is the time, *ρ* is the fluid density, and *μ*_eff_ is the effective viscosity. The wall-adaption local eddy-viscosity (WALE) model was selected as the subgrid scale (SGS) model for modeling the effects of unsolvable scale of turbulence on solvability turbulence. The coupling between the pressure and velocity used the scheme of PISO, owing to its distinctive advantages in solving the transient problems. The user-defined inlet velocity was specified normal to the boundary planes of nostrils, and the static pressure was set to be zero at the outlet. In addition, the no-slip boundary condition was implemented on all the solid walls.

## 3. Results and Discussion

### 3.1. Experimental Validation

**Figure 2:**
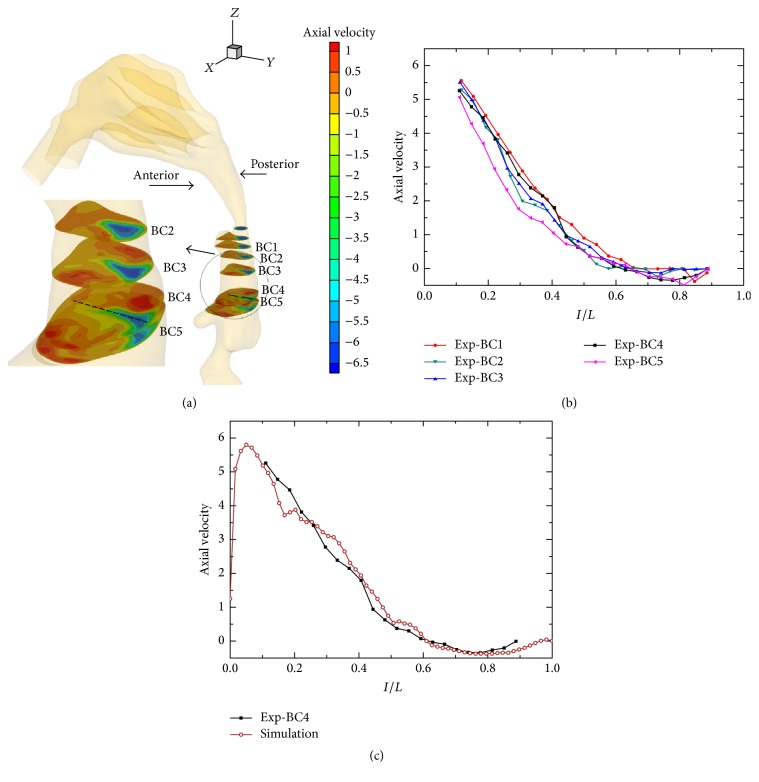
The experimental validation. (a) The axial velocity contours for obstructed HUA model. The dash line is the location of LDA measurement. (b) The measured axial velocity profiles in five cross-sections. (c) The comparison between measured and calculated velocity profile. Here, the axial velocity means the velocity in the *z*-axis direction.

Firstly, the comparison between the measured and calculated results in the center-line of cross-section was performed to validate the reliability of present CFD method. In the experiments, the velocity profiles inside HUA model were measured by the LDA. The measuring cross-sections (BC1–BC5) were indicated in Figure 2(a). Owing to the narrowing of obstructed HUA, only one measuring center-line in the cross-section BC4, which was shown in dash line, was chosen for comparison with the simulation results. The measurement was started from the posterior side to the anterior side of HUA with horizontal movement. In the simulations, the airflow field was solved by the software of CEM-CFD, which simultaneously adopted LES turbulent model to reveal the transitional/turbulent unsteady, separated, or vortical flows. The respiratory intensity in experiments and simulations was set to be constant with *Q* = 16.8 L/min, which corresponded to a constant inlet velocity of *u* = 0.8 m/s that was calculated according to the respiratory intensity and the nostril cross-sectional area.

Figure 2(b) shows the measured velocity profiles in the obstructed HUA model. The axial velocity distributions in BC1–BC5 experience a similar trend, which both is higher near the posterior side due to the strong effect of “pharyngeal jet” and becomes weak and negative near the anterior wall owing to the reversed flow effect. The maximal velocity in five cross-sections BC1–BC5 can reach approximate 5.1~5.6 m/s.  Figure 2(c) illustrates the comparison between the measured and calculated data in the center-line of cross-section BC4, where the radial distance has been normalized. In general, the agreement of velocity profiles between them is good. Although the velocity very near to the wall cannot be captured due to the limitation of LDA technique, the velocity profiles and values are almost the same as the calculation in entire measurable region, which identified that the present CFD method can be used to study airflow dynamics in the obstructed HUA model.

### 3.2. Grid Independence Test

**Figure 3:**
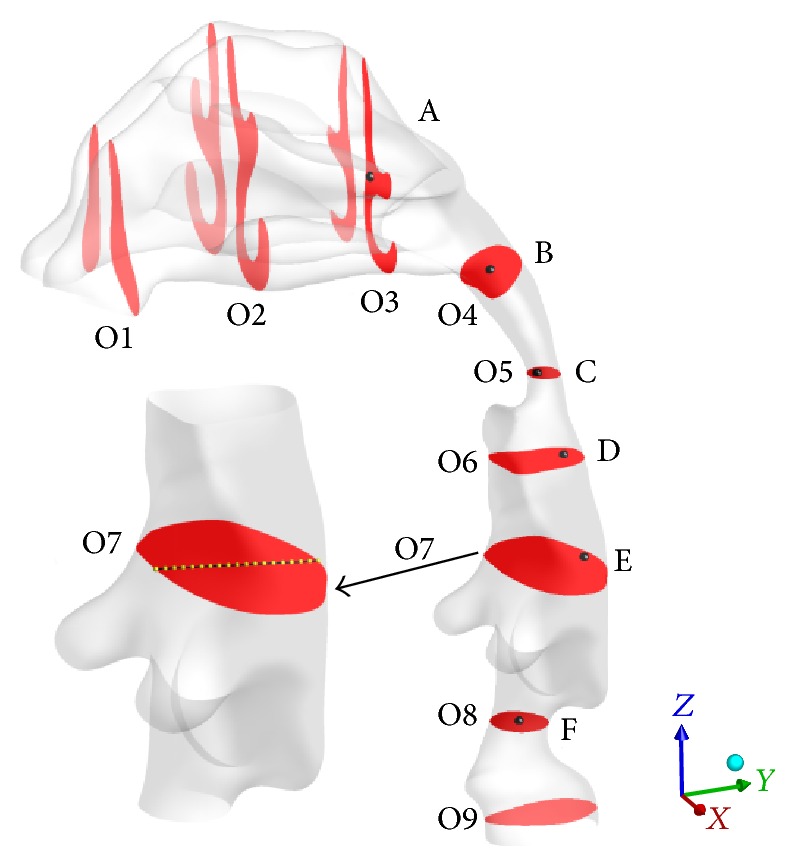
Characteristic cross-sections and points in the obstructed HUA model.

To verify the grid independence, three different gird sizes (1862545, 1283535, and 834353 cells) were chosen to conduct the calculations under the respiration intensity of 16.8 L/min at *t* = 1.25 s, in which the maximal inspiration occurs in the respiration process. As shown in Figure 3, nine characteristic cross-sections (O1–O9), which represent the typical locations of nasal valve, anterior turbinates, posterior turbinates, oropharynx, pharynx, tip of epiglottis, middle of epiglottis, laryngeal, and trachea, and six characteristic points (A–E) were chosen in the obstructed HUA model. Owing to the narrowing of HUA, the measuring center-line in the characteristic cross-section O7, which is shown in dash line in Figure 3, was adopted to execute the grid independence test.

**Figure 4:**
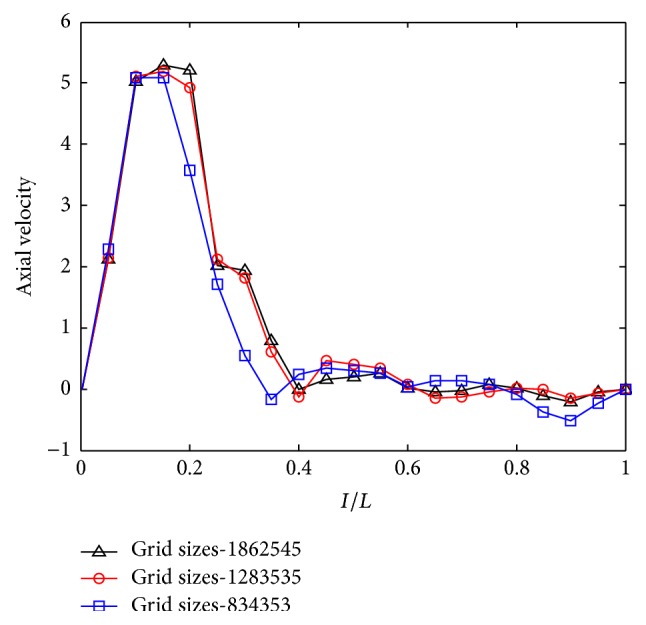
The grid independence test under *Q* = 16.8 L/min.

From Figure 4, one can find that the agreement of axial velocity for three gird sizes is excellent from the posterior wall to the center of jet region. However, the axial velocity of the grid size of 834353 cells is much smaller than that of the other two grid sizes from the center of jet region to jet margin. From the jet margin to anterior wall, the axial velocity has little difference for all three grid sizes. In general, the discrepancy of axial velocity between 1283535 and 1862545 cells is much smaller comparing to that of 834353 cells. It seems that 1283535 cells can satisfy the request of calculations and was chosen in the rest of simulations.

### 3.3. Airflow Dynamics in the Obstructed HUA

In the simulations, the respiration intensities of *Q* = 16.8 L/min (slight breathing), 30 L/min (moderate breathing), and 60 L/min (severe breathing) were conducted under continuous inspiration and expiration conditions. After two respiratory cycles, the velocity and pressure fields in circular breathing were obtained.

**Figure 5:**
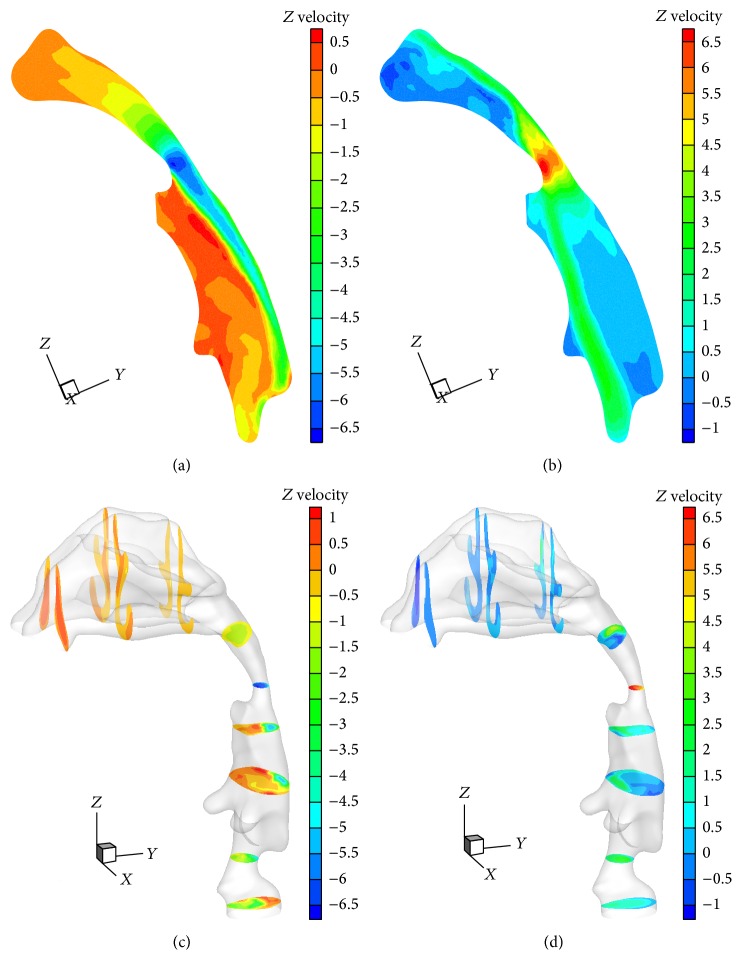
Instantaneous velocity contours in sagittal planes ((a), (b)) and in axial plane ((c), (d)). ((a), (c)) Inspiration; ((b), (d)) expiration.

Figures 5(a) and 5(b) present the instantaneous velocity contour in sagittal plane between posterior turbinates and laryngeal in circular breathing at *Q* = 16.8 L/min and *t* = 1.25, 3.75 s, in which the maximal inspiration and expiration occur in the respiratory cycle. Based on the definition of Re = *ρu*_ave_ *d*_eff_/*μ*_eff_, the inlet Reynolds numbers in inspiration and expiration are 372 and 512, respectively. Here, *ρ* is the air density, *u*_ave_ is the average inlet velocity in a half cycle, *d*_eff_ is the effective inlet size, and *μ*_eff_ is the effective air viscosity. During inspiration, the air flows from the nostrils to the trachea. The instantaneous velocity gradually increases from the posterior turbinates to the pharynx and finally reaches the peak in the pharynx. There exists a jet flow in the downstream region of pharynx, due to the narrowing occurring in the pharynx. During expiration, the air flows from the trachea to the nostrils. Likewise, the maximal velocity of airflow occurs in the pharynx. A jet flow is also found in the downstream region of pharynx. The instantaneous velocity contours in axial planes in inspiration and expiration at *Q* = 16.8 L/min and *t* = 1.25, 3.75 s are displayed in Figures 5(c) and 5(d). Due to the sudden expansion of airway after the nostrils, the velocities in the anterior turbinates and posterior turbinates are much less than any other regions in inspiration. However, the jet flow leads to much larger velocity in the posterior turbinates than that in the anterior turbinates in expiration. The obvious flow separation and back flow were observed after the pharynx in inspiration due to the strong effect of jet flow. Likewise, the jet flow effect causes strong flow separation and back flow in the oropharynx and turbinates in expiration.

**Figure 6:**
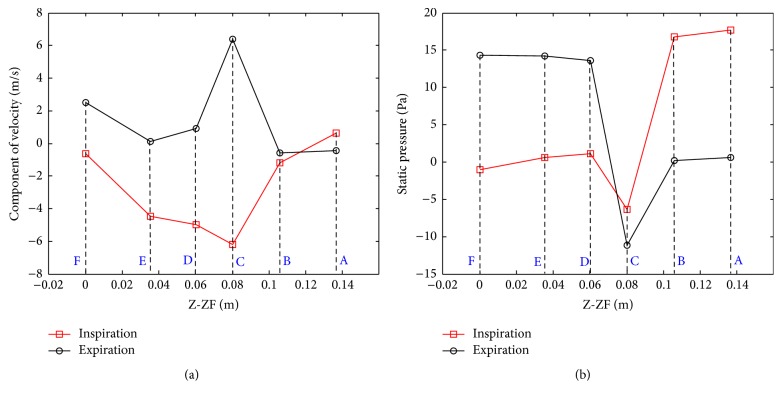
Instantaneous velocity and static pressure at characteristic points. (a) Velocity; (b) static pressure.

Six characteristic points before and after the pharynx are chosen to analyze the variation of velocity and static pressure at *Q* = 16.8 L/min and *t* = 1.25, 3.75 s.  Figure 6(a) displays the instantaneous velocity components (*u*_*y*_ and *u*_*z*_) at these characteristic points. It can be noted that the largest amplitudes of instantaneous velocity appear at point C, which is located in the characteristic cross-section O5, in both inspiration and expiration processes. It is about 6.20 m/s at C in inspiration, slightly smaller than that of 6.40 m/s in expiration. The static pressures at six characteristic points are shown in Figure 6(b). In inspiration, the static pressure decreases from A to C, and it reaches a minimum of −6.35 Pa at the narrowest cross-section O5, where the airflow attains its maximal velocity. The changing static pressure from C to E usually creates a recirculation zone (flow reversal) due to the adverse pressure gradient. The expiration process causes occurrence of opposite phenomenon compared to the inspiration process except for the sharp drops of static pressure at C. During expiration, the minimal static pressure is nearly −11.16 Pa, much lower than that in inspiration. Apparently, there are two large pressure differences appearing before and after a flow constriction at C, indicating that the obstruction arises at pharynx, which also verifies the approach to locating the airway collapse using pressure difference between the sensors [18].

**Figure 7:**
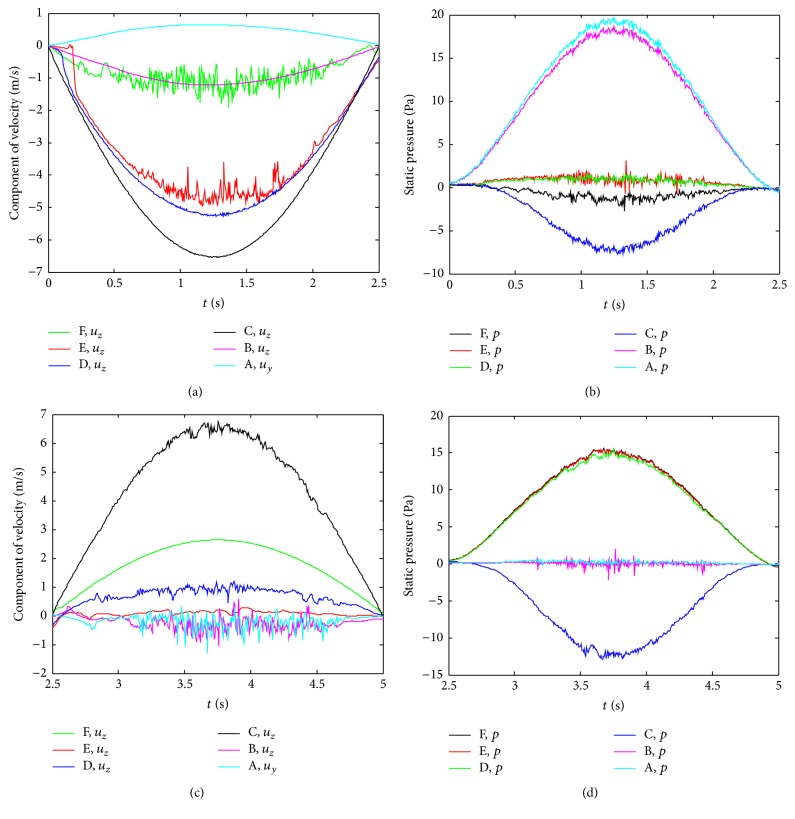
Instantaneous velocity components ((a), (c)) and static pressure ((b), (d)) at characteristic points. ((a), (b)) Inspiration; ((c), (d)) expiration.

Figure 7 illustrates the variations of two instantaneous velocity components (*u*_*y*_ and *u*_*z*_) and static pressure at six characteristic points at *Q* = 16.8 L/min in whole circular breathing process. It can be seen from Figures 7(a) and 7(c) that the amplitudes of the velocity are very different; however, all of them almost experience sinusoidal waves at these characteristic points, owing to the periodic sinusoidal boundary conditions imposed in the inlets. During inspiration, the velocities at A and B fluctuate weakly, indicating a transition from laminar to turbulent flow there. C is located at the cross-section of pharynx; the greatest velocity occurs there with less fluctuation. The reduction of cross-sectional area between the nasopharynx and oropharynx generates a jet downstream of the restriction. Since D is still inside the region of jet flow, the velocity distribution experiences less fluctuation there. E is located in the area of jet reduction and back flow expansion; the fluctuation of the velocity gradually increases because of the strong turbulent and back flow there. With the constriction of larynx, the intensity of turbulent rapidly strengthens; there also exists substantially fluctuation of the velocity at F resulting from the violent low Reynolds turbulent and reversed flow. It can be confirmed that the airflow in the pharynx, larynx, and trachea is turbulent and unsteady. During expiration, due to the jet flow and the complication of epiglottis, the monitoring points generate varying degrees of fluctuation with the exception of F, revealing that the degree of monolithic turbulence in expiration is larger than that in inspiration.

The process of human respiration depends on the expansion and contraction of pleural, and the static pressure components at characteristic points are presented in Figures 7(b) and 7(d). Since the pressure in the nasal cavity is less than that of the atmosphere, A and B exhibit positive pressures in inspiration. Due to the contraction of pharynx, the static pressure at C turns negative and varies between 0 and −10 Pa. With the widening of airway, the static pressure from D to F gradually arises and becomes positive. Correspondingly, a recirculation zone is produced with the negative pressure drop from pharynx to downstream region. In expiration, the static pressures at D–F are almost coincident. Likewise, the highest negative pressure appears at C, and the static pressure fluctuates and tends to 0 at both A and B. Meanwhile, a similar recirculation zone is created from pharynx to turbinates.

**Figure 8:**
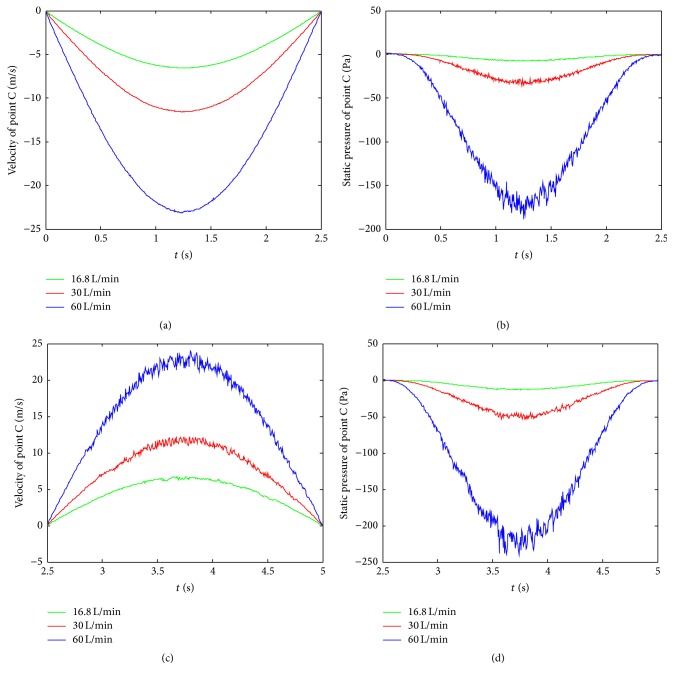
Instantaneous velocity ((a), (c)) and static pressure ((b), (d)) at point C under *Q* = 16.8 L/min, 30 L/min, and 60 L/min. ((a), (b)) Inspiration; ((c), (d)) expiration.

Owing to the particular contraction of pharynx, the variation of instantaneous velocity and static pressure at C are chosen and calculated under three typical respiratory intensities, namely, *Q* = 16.8 L/min (slight breathing), 30 L/min (moderate breathing), and 60 L/min (severe breathing), which are displayed in Figure 8. During inspiration, the trends of the velocity are similar, whose profiles all shape sinusoidal curves, although their amplitudes are very different. It can be observed that the velocities arise rapidly as the increase of respiratory intensity. During expiration, the volatilities of velocity become more obvious with the raise of respiration intensities due to the jet flow effect. The amplitudes of velocity in same respiratory intensity are approximate in both inspiration and respiration. From Figures 8(b) and 8(d), it is found that amplitude of static pressure under *Q* = 60 L/min respiratory intensity is almost 5 times that under *Q* = 30 L/min and 24 times that under *Q* = 16.8 L/min, which indicates that the respiratory intensity significantly affects the instantaneous velocity and static pressure. The fluctuations of static pressure rise fast with increasing of respiratory intensity in both inspiration and expiration, since the larger respiratory intensity may cause stronger turbulent flow inside the HUA model.

## 4. Conclusion

In this work, the flow characteristics in the realistic suffering HUA resulted from pharyngeal collapse were numerically investigated based on the CT-CFD approach. The 3D anatomically accurate HUA model was reconstructed from CT-scan images of a Chinese male patient. The CFD solver with the LES method was applied to simulate the laminar-transitional-turbulent airflow in the obstructed HUA model. In addition, the LDA measurements for airflow field in the suffering HUA model were executed to validate the reliability of LES approach. In the calculations, the continuous circular breathing was accomplished by altering the boundary conditions, and the airflow field and pressure field were computed on the basis of real circular inspiration and expiration processes. The instantaneous velocity and static pressure in several selected characteristic cross-sections and points are discussed at slight breathing, moderate breathing, and severe breathing. The results reveal that airflow in the HUA model is an unsteady transitional/turbulent flow with low Reynolds number. The strong flow injection phenomenon caused by narrowing of pharynx is found in both inspiration and expiration. The strong flow separation and back flow inside HUA are also observed due to the vigorous jet flow effect in the pharynx. In addition, it is found that the respiratory intensity has great effect on the airflow dynamics. The analysis for airflow field in the suffering HUA model could come up with great assistance for the treatment of obstructive respiratory disease. What is worth mentioning is that the present CT-CFD based computational method is applicable but not limited in modeling the flows in the circulatory and respiratory systems; it can also be applied to study the flows in the circulatory and reproductive systems, that is, sperm swimming in fertilization.
